# The influence of conjugation variables on the design and immunogenicity of a glycoconjugate vaccine against *Salmonella* Typhi

**DOI:** 10.1371/journal.pone.0189100

**Published:** 2017-12-29

**Authors:** M. Arcuri, R. Di Benedetto, A. F. Cunningham, A. Saul, C. A. MacLennan, F. Micoli

**Affiliations:** 1 GSK Vaccines Institute for Global Health (GVGH), Siena, Italy; 2 University of Birmingham, Edgbaston, Birmingham, United Kingdom; 3 Jenner Institute, Nuffield Department of Medicine, University of Oxford, Oxford, United Kingdom; New York State Department of Health, UNITED STATES

## Abstract

In recent years there have been major efforts to develop glycoconjugate vaccines based on the Vi polysaccharide that will protect against *Salmonella enterica* Typhi infections, particularly typhoid fever, which remains a major public health concern in low-income countries. The design of glycoconjugate vaccines influences the immune responses they elicit. Here we systematically test the response in mice to Vi glycoconjugates that differ in Vi chain length (full-length and fragmented), carrier protein, conjugation chemistry, saccharide to protein ratio and size. We show that the length of Vi chains, but not the ultimate size of the conjugate, has an impact on the anti-Vi IgG immune response induced. Full-length Vi conjugates, independent of the carrier protein, induce peak IgG responses rapidly after just one immunization, and secondary immunization does not enhance the magnitude of these responses. Fragmented Vi linked to CRM_197_ and diphtheria toxoid, but not to tetanus toxoid, gives lower anti-Vi antibody responses after the first immunization than full-length Vi conjugates, but antibody titres are similar to those induced by full-length Vi conjugates following a second dose. The chemistry to conjugate Vi to the carrier protein, the linker used, and the saccharide to protein ratio do not significantly alter the response. We conclude that Vi length and carrier protein are the variables that influence the anti-Vi IgG response to immunization the most, while other parameters are of lesser importance.

## Introduction

Typhoid fever remains a major public health concern in low-income countries and affects millions of people each year [1]. Vaccination is considered the most promising strategy for the control of the disease [2]. Antibodies directed against the Vi antigen, which forms a polysaccharide (PS) capsule around *Salmonella enterica* serovar Typhi (*S*. Typhi), can offer protection and Vi PS is currently licensed as a vaccine against typhoid fever [3]. Being a T-independent antigen, the Vi PS is not immunogenic in infants and is only licensed for children over two years of age [4, 5]. Recent years have seen major efforts to develop glycoconjugate vaccines against *S*. Typhi [6, 7]. Conjugation of Vi PS to a carrier protein can effectively convert the T-independent PS antigen into a T-dependent antigen. This can enhance the memory response and allow protective immunity to develop in children and infants, as well as adults [8].

The synthesis of glycoconjugate vaccines requires the covalent linkage between the saccharide and the carrier protein. Different conjugation methods can be used, all following two main approaches: random linkage along the PS chain, or selective attachment at the terminal end of the sugar moiety. Spacer molecules are often introduced between the saccharide and the protein to reduce steric hindrance and facilitate interaction between both moieties [8]. The conjugation method and linker used together with saccharide chain length, carrier protein, saccharide to protein ratio, and saccharide structure, are conjugation variables that can affect the magnitude, quality and persistence of the antibody response elicited [8, 9].

We developed a Vi-CRM_197_ conjugate vaccine [10, 11], that has been tested in Phase 1 and 2 trials in Europe [12] and endemic countries [13]. Vi-CRM_197_ was considerably more immunogenic than unconjugated Vi, and able to induce specific antibody responses in infants [13]. However, a second injection of conjugate given 8 weeks apart had no incremental effect on antibody levels and the persistence of the anti-Vi response was similar to that induced by unconjugated Vi [13]. Similarly, an absence of boosted antibody response after vaccination with Vi-TT was shown in another study [14].

With the aim of improving vaccine design, in this study we investigate the impact of different conjugation variables on the immunogenicity of glycoconjugate vaccines against *S*. Typhi, by altering just one parameter in each candidate used, whilst keeping the others constant. Whilst some studies have already investigated the influence of such parameters on the immunogenicity of Vi glycoconjugate vaccines [10, 11, 15–20], they have typically focused on the effects of one parameter only or have changed multiple parameters within a single conjugate vaccine.

Our findings show that only a small number of parameters influence the immune response to the vaccines tested. These results will help guide the design of Vi glycoconjugate vaccines with an enhanced potential to protect.

## Materials and methods

### Purification of Vi PS

Vi PS was purified at GVGH from *Citrobacter freundii* NVGH 328 as previously described [21]. The lot used for this study contained 0.3% protein (by micro BCA), 0.001% nucleic acid (by picogreen) (weight to weight respect to the sugar) and endotoxin level of 1.16 EU/ μg of sugar (by LAL test). O-acetylation level was > 90% as detected by ^1^H NMR and average molecular weight (avMW) was of 165 kDa, as estimated by HPLC-SEC analysis (TSK gel 3000 PW_XL_ column) using dextrans as standards.

### Proteins used for conjugation

CRM_197_, DT and TT were obtained from GSK Vaccines S.r.l., Siena. Tetanus toxoid was purified by gel filtration through Sephacryl S300 (GE Healthcare) equilibrated in 0.15 M NaCl, 10 mM NaH_2_PO_4_, pH 7.2. The fractions corresponding to the monomeric MW of TT were pooled and used for conjugation.

### Chemicals

The following chemicals were used in this study: adipic acid dihydrazide (ADH), oxalildihydrazide (ODH), pimelic acid dihydrazide (PDH), succinic dihydrazide (SDH) *N*-(3-dimethylaminopropyl)-*N*’-ethylcarbodiimide hydrochloride (EDAC), *N*-hydroxisuccinimide (NHS), 4-(4,6-dimethoxy-1,3,5-triazin-2-yl)-4-methylmorpholinium chloride (DMT-MM), succinimidil 3-(bromoacetamido) propionate (SBAP), cystamine dihydrochloride, sodium cyanoborohydride (NaBH_3_CN), 4-morpholine ethanesulfonic acid (MES), sodium chloride (NaCl), sodium hydroxide (NaOH), hydrochloride acid 37% (HCl), dimethyl sulfoxide (DMSO), sodium acetate (AcONa), sodium phosphate monobasic monohydrate (NaH_2_PO_4_^.^H_2_O), 2,4,6-trinitrobenzenesulfonic acid (TNBS) [Sigma], dithiothreitol (DTT) [Invitrogen], phosphate buffered saline tablets (PBS) [Fluka], ethylenediaminetetraacetic acid (EDTA) disodium salt [Merk], acetonitrile (CH_3_CN) [Prolabo], NHS-PEG_4_-N_3_ [Thermo & Fisher], click easy BCN NHS ester I alkyne linker [Berry & Associate].

### Method for making fragmented Vi (fVi) and its characterization

Vi, freeze dried as the sodium salt, was solubilized in water and H_2_O_2_ was added to give a final concentration of 2.5 mg/mL Vi and 5% (wt/v) H_2_O_2_ in water. The mixture was heated at 80±0.5°C for 2h. The mixture was then injected into a Hiscreen Capto Q [GE Healthcare] column (4.7 mL of resin loading up to 100 mg of fragmented Vi mixture) equilibrated with buffer A and populations of different average size were separated using a gradient step method. NaH_2_PO_4_ 20 mM pH 7.2 and NaH_2_PO_4_ 20 mM NaCl 1M pH 7.2 were used as buffer A and B respectively. Pools at average size Vi of 8.6 and 43 kDa were eluted at 25 and 37% of buffer B respectively. Each pool was desalted on a Sephadex G-25 column [GE Healthcare] equilibrated with water. The average size of the fragmented Vi pools was determined by HPLC-SEC equipped with a TSK gel 3000 PW_XL_ column and a TSK gel PW_XL_ guard column (Tosoh Bioscience). Dextrans (5, 25, 50, 80, 150 kDa) were used as standards (Sigma Aldrich). The mobile phase was 0.1 M NaCl, 0.1 M NaH_2_PO_4_, 5% CH_3_CN, pH 7.2, at the flow rate of 0.5 mL/min (isocratic method for 30 min). HPAEC-PAD was used to measure Vi content [10, 21]. ^1^H NMR was used to verify Vi identity and confirm O-acetylation levels were >60% [10, 21].

### Synthesis of full-length and fVi conjugates

#### Use of different carrier proteins: Vi activation with EDAC/NHS followed by conjugation to the protein derivatised with ADH linker

With fVi avMW 43 kDa, the following procedure was used to prepare conjugates. Polysaccharide was solubilized in 100 mM MES pH 6 at a concentration of 50 mg/mL. NHS and then EDAC were added to have 0.33 M NHS and EDAC/Vi repeating units molar ratio of 5. After the reaction was mixed at room temperature for 1h, the protein previously derivatized with ADH [10, 21], was added to give a Vi concentration of 7.8 mg/mL in 20 mM MES, pH 6 and mixed at room temperature for 2h. For full length Vi, the PS concentration in the EDAC/NHS activation step was reduced to 4.2 mg/mL and to 1.7–3.5 mg/mL in the conjugation step in order to avoid gel formation. Different ratios of Vi to protein were used: 1:1, 2:1 or 1:2 in weight.

Full-length Vi-CRM_197_ conjugates were purified by tangential flow filtration by using a 300k membrane (Sartocon Slice Cassette 200 cm^2^ PES). Twenty cycles of diafiltration against 1M NaCl 20 mM NaH_2_PO_4_ pH 7.2 and subsequently twenty cycles of diafiltration against 20 mM NaH_2_PO_4_ pH 7.2 (Pin 2.0 bar; Pout 0.2 bar; permeate flow rate = 30–33 mL/min) were performed. For full-length Vi-DT conjugate purification was performed with a 100k membrane (Hydrosart 200 cm^2^ in stabilized cellulose). Full-length Vi-TT conjugate and fVi conjugates were purified by size exclusion chromatography on a 1.6 cm x 60 cm Sephacryl S300 column or 1.6 cm x 60 cm Sephacryl S100 HR column respectively [GE Healthcare] eluted at 0.5 mL/min in PBS. Fractions at higher MW that did not overlap free PS and free protein run on the same column in the same conditions were collected.

Activated Vi (with EDAC/NHS) was not isolated before protein addition, but a fraction of the mixture was sampled in process and characterized for quantifying the % of activated Vi repeating units (molar ratio % of NHS/Vi repeating units). The sample was desalted by PD10 column (SephadexTM G-25M, GE Healthcare) against HCl 55 ppm and analyzed by ion pair HPLC-RP for NHS quantification and by HPAEC-PAD for Vi PS quantification. For quantification of NHS ester groups introduced on Vi PS, samples were eluted on a C18 column (Phenomenex, Gemini-NX 5 μ) with 80% 10 mM TBABr, 0.17% NH_4_OH, 20% ACN in isocratic condition with a flow rate of 1 mL/min. Eluent pH allowed ester-NHS groups hydrolysis and formation of N-hydroxysuccinimidate anion that was detected at 260 nm eluted as ion pair with TBA. Calibration curve was built using NHS as standard in the range 3–50 nmol/mL.

#### fVi-CRM_197_ conjugates differing for conjugation chemistry

fVi-CRMODH, fVi-CRMSDH, fVi-CRMPDH: fVi activation with EDAC/NHS followed by conjugation to the protein derivatised with linkers of different length. CRM_197_ was derivatized with ODH, SDH or PDH, as previously described for ADH [10, 21]. Conjugation step with fVi avMW 43 kDa was performed as described for CRMADH, with a fVi to protein ratio 1:1 in weight, but increasing Vi concentration to 15 mg/mL to have all the protein conjugated after 2h mixing at room temperature. The conjugates were purified by size exclusion chromatography on a 1.6 cm x 60 cm Sephacryl S100HR column eluting at 0.5 mL/min in PBS.

fVi(ADH)-CRM_197_: fVi randomly derivatised with ADH linked to CRM_197_ after activation of protein COOH groups with EDAC/NHS. Fragmented Vi avMW 43 kDa was solubilized in100 mM MES pH 6 at a concentration of 15 mg/mL. NHS and then EDAC were added to have 0.1 M NHS and EDAC/Vi repeating units molar ratio of 5. After the reaction was mixed at room temperature for 1h, ADH was added (molar ratio ADH to Vi repeating units of 1.5). The mixture was mixed at room temperature for 2h and then desalted by PD10. No crosslinking was confirmed by HPLC-SEC and 22% repeating units resulted activated by TNBS colorimetric method. For the step of conjugation, CRM_197_ was diluted with 600 mM MES pH 6 at a concentration of 15.5 mg/mL. NHS and then EDAC were added to have 0.1 M NHS and EDAC/COOH groups molar ratio of 5. After the reaction was mixed at room temperature for 1h, fViADH was added to have a fVi concentration of 10 mg/mL and with a Vi to protein ratio 1:2 in weight in MES 100 mM pH 6. The reaction was mixed at room temperature for 2h. The conjugate was purified by size exclusion chromatography on a 1.6 cm x 60 cm Sephacryl S100HR column eluting at 0.5 mL/min in PBS. It was verified by HPLC-SEC that no protein aggregation happened in the reaction conditions used.

fVi(DMT-MM)-CRMADH: fVi randomly activated with DMT-MM linked to CRM_197_ after its derivatization with ADH. Fragmented Vi with an avMW 43 kDa was solubilized in NaH_2_PO_4_ 100 mM pH 7 to have a fVi concentration of 10 mg/mL and DMT-MM was added with a molar ratio of 5 respect to Vi repeating units. The reaction proceeded at room temperature for 10 minutes and CRMADH was then directly added to the solution to have a fVi to protein ratio 1:1 in weight with a fVi concentration of 3.8 mg/mL. After mixing at room temperature for 2h, the conjugate was purified by size exclusion chromatography on a 1.6 cm x 60 cm Sephacryl S100 HR column eluting at 0.5 mL/min in PBS.

fViADHN_3_CRMalkyne: fVi linked to CRM-alkyne after random derivatization with azido groups. Fragmented Vi avMW 43 kDa was randomly activated with ADH as previously described. Derivatised fVi (25% fVi repeating units activated according to TNBS) was then mixed with the linker NHS-PEG_4_-N_3_ (25 mg/mL in DMSO) in NaH_2_PO_4_ 100 mM pH 7.2 at a concentration of 3.6 mg/mL fVi and with a moral ratio azido linker to NH_2_ groups on fVi 2:1. The reaction was mixed at room temperature for 4h and the product purified by PD10 eluting with NaH_2_PO_4_ 10 mM pH 7.2. Ninety % NH_2_ groups introduced on fVi through ADH resulted derivatised, as verified by TNBS method.

CMR_197_ was diluted in PBS and click easy BCN NHS ester I alkyne linker (10 mg/mL in DMSO) was added (molar ratio linker to lysines on CRM_197_ of 0.76) resulting in a protein concentration of 8.6 mg/mL. After mixing at room temperature for 5h, the mixture was purified by PD10 eluting with PBS. An average of 12 linkers resulted introduced per CRM_197_ molecule by MALDI MS [10].

Conjugation was performed in PBS with a final concentration of protein at 12 mg/mL and a molar ratio of azido to alkyne groups 6:1. The solution was mixed at room temperature overnight and the resulting conjugate purified by hydrophobic interaction chromatography on a Phenyl HP column [GE Healthcare], loading 300 μg of protein for mL of resin in 50 mM NaH_2_PO_4_ 3M NaCl pH 7.2. The purified conjugate was eluted in water and the collected fractions were dialysed against PBS.

fVi_s_(ADH)CRM_197_: fVi activated with ADH at the reducing end and linked to CRM_197_ after activation of COOH groups on the protein with EDAC/NHS. Fragmented Vi avMW 8.6 kDa was dissolved in AcONa 20 mM pH 4.5 at a concentration of 30 mg/mL. Reductive amination was performed by adding ADH and NaBH_3_CN (respectively 6 and 17 fold molar excess respect to fVi chains). The reaction proceeded for 3 days at 30°C. The solution was then diluted in NaCl 3 M and desalted twice by PD10. 95% of fVi chains resulted activated by TNBS.

Fragmented Vi derivatised with ADH was added to CRM_197_ activated with EDAC/NHS as previously described, in order to obtain a fVi and CRM_197_ concentration respectively of 30 and 10 mg/mL and a molar ratio of fVi chains to CRM_197_ of 20:1. The reaction proceeded for 2 hours at room temperature.

Conjugate was purified by size exclusion chromatography on a 1.6 cm x 60 cm Sephacryl S100 HR column eluting at 0.5 mL/min in PBS.

fVi_s_SHCRMSBAP: fVi activated with cysteine at the reducing end and conjugated with CRM_197_ previously derivatised with SBAP. CRM_197_ was solubilized in NaH_2_PO_4_ 100 mM EDTA 1 mM pH 8.0 (5.1 mg/mL); SBAP was added (0.3 mg/mL, molar ratio SBAP/lysine groups on CRM_197_ = 0.3) after being solubilized in DMSO (final DMSO concentration of 4% v/v). The mixture was stirred for 3h at room temperature, and then purified by PD10 column eluting with NaH_2_PO_4_ 100 mM EDTA 1 mM pH 7.0. An average of 9 linkers was introduced per CRM_197_, as determined by MALDI-MS [10].

Fragmented Vi avMW 8.6 kDa was solubilized in NaH_2_PO_4_ 100 mM pH 7.0 (20 mg/mL) and then cystamine (112.5 mg/mL, cystamine/fVi (w/w) = 5.6) and NaBH_3_CN (50 mg/mL, NaBH_3_CN/fVi (w/w) = 2.5) were added. The mixture was stirred for 5 days at 30°C and then desalted after diluting the sample with NaCl 6 M by PD10 column. Cystamine disulfide bond was reduced by mixing derivatised fVi at a concentration of 20 mg/mL with DTT 100 mM in NaH_2_PO_4_ 100 mM EDTA 5 mM for 1h at room temperature. The derivatized fVi was purified by desalting on PD10 against 10 mM NaH_2_PO_4_ 5 mM EDTA pH 7.5. Seventy % fVi chains resulted activated with cystamine, according to TNBS, and reduction with DTT was complete, as verified by Ellman colorimetric method.

Conjugation was performed in NaH_2_PO_4_ 100 mM EDTA 1 mM pH 7.2 with protein concentration of 12 mg/mL and using a molar ratio of fVi chains to CRM_197_ of 20 to 1. After mixing 3h at room temperature the conjugate was purified by hydrophobic interaction chromatography on a Phenyl HP column, as previously described.

#### Characterization of the conjugates generated

Purified conjugates were characterized by HPAEC-PAD for total Vi content [10], micro BCA for total protein content, HPLC-SEC for determining avMW distribution of the conjugate and to assess the amounts of free protein (fluorescence emission) and free saccharide (refractive index) for fVi avMW 8.6 kDa. For conjugates prepared with fVi avMW 43 kDa as for full-length Vi conjugates, free saccharide was estimated by Capto Adhere/HPAEC-PAD method [22].

### Immunization of mice and assessment of antibody responses

Female 10 weeks or 5 weeks old outbred CD-1 mice were purchased from Charles River Laboratory. All animal protocols were approved by the local animal ethical committee (GSK Animal Welfare Body) and by the Italian Minister of Health in accordance with Italian law.

In all the immunogenicity studies performed eight mice per group were injected subcutaneously into the back two times, at 4–5 week intervals, with 200 μL/dose of conjugated Vi. Antigens were diluted in 0.9% w/v saline solution without adjuvant. Mice were bled and sera collected before first immunization (day 0), two weeks after the first immunization, the day of the second immunization and two weeks after the second immunization. Anaesthesia, tielamine/zolazepam (10–40 mg/kg p.v.) and xilazina (0.4–4 mg/kg p.v.), were administered via the intraperitoneal route (max 0.5 ml/mouse) before final bleeding and euthanasia. Carbon dioxide or cervical dislocation in pre-anesthetized mice were used as methods of euthanasia. The condition of animals was monitored twice per day. No signs of suffering or inflammation at the site of injection were reported after subcutaneous immunizations. Humane endpoints were defined and approved by the Animal Welfare Body and personnel was trained to respond promptly after the detection of adverse events. Animals with severe signs of illness would have been sacrificed under the approval and responsability of the designated veterinary, research project and animal facility. For moderate clinical signs, the use of analgesics was avoided because of their potential immunomodulatory and anti-inflammatory effects.

Serum IgG levels against Vi and carrier proteins were measured by ELISA using the method previously described [11].

Statistical and graphical analysis was performed using GraphPad Prism 6 software. The non-parametric Mann-Whitney test and Kruskal-Wallis analysis with Dunn’s test for post hoc analysis were used to compare respectively two or multiple groups. Wilcoxon matched-pairs signed rank two-tailed test was used to compare results from the same group at different time points.

## Results

### Vi chain length and the carrier protein used affect anti-Vi IgG responses induced by Vi-conjugates

Three different proteins (DT, TT and CRM_197_) were used as carriers for full-length (165 kDa) and fVi with an average MW of 43 kDa. Carboxylic groups along the PS chain were randomly activated with carbodiimide and NHS and linked to the carrier protein, previously derivatised with ADH as a spacer. CRM_197_ was compared to DT and TT, two other proteins commonly used in the production of glycoconjugate vaccines [8], including Vi conjugate vaccines [6, 7].

**Fig 1:**
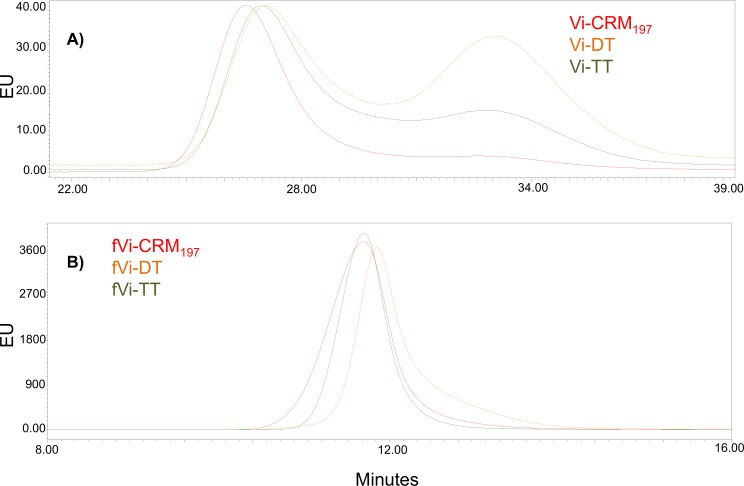
A) HPLC-SEC profiles (fluorescence emission detection) of Vi-CRM_197_ (red), Vi-DT (orange) and Vi-TT (green). TSK gel 6000–5000 PW columns, NaCl 0.1 M NaH_2_PO_4_ 0.1 M ACN 5%, pH 7.2; flow 0.5 mL/min; V_tot_ 49.004 min; V_0_ 24.382 min. B). HPLC-SEC profiles of fVi-CRM_197_ (red), fVi-DT (orange), fVi-TT (green). TSK gel 3000 PWxl column; NaCl 0.1 M NaH_2_PO_4_ 0.1 M ACN 5%, pH 7.2; flow rate 0.5 mL/min; V_tot_ 23.326 min, V_0_ 10.663 min.

The conjugation protocol was optimized using CRM_197_, both for full-length and shorter Vi chains. The same procedures were then applied to DT and TT. On average, 12 and 24 ADH linkers were introduced on DT [15] and TT respectively, compared to 6 linkers on CRM_197_, as quantified by MALDI-TOF analysis (Table 1). Corresponding conjugates were synthesised with a PS to protein weight to weight (w/w) ratio of 1:1. HPLC-SEC profiles (Fig 1) of the resulting conjugates showed that Vi-DT and Vi-TT were characterized by two main conjugate populations of differing average MW, while the Vi-CRM_197_ conjugate only had the population of higher MW. Table 1 lists main characteristics of the conjugates obtained, without separating any populations characterized by different MW.

**Table 1:** Characterization of full-length and fragmented Vi conjugates by using different carrier proteins.

Conjugate	Average number ADH linkers per protein	% PS RU activated with NHS	Total PS to protein w/w ratio	% free PS	% free protein
Vi-CRM_197_	**6**	20.5	1.4	6.7	nd
Vi-DT	**12**	23.2	3.1	6	nd
Vi-TT	**24**	18.9	1.3	34.2	nd
fVi-CRM_197_	**5**	21.3	0.5	<15	nd
fVi-DT	**12**	21.3	0.86	<20	nd
fVi-TT	**24**	21.3	0.40	<6.8	nd

nd: not detectable; RU: repeating units.

Both for full-length and fragmented Vi, the DT conjugates were characterized by higher Vi to protein w/w ratio than CRM_197_ and TT ones (Table 1).

**Fig 2:**
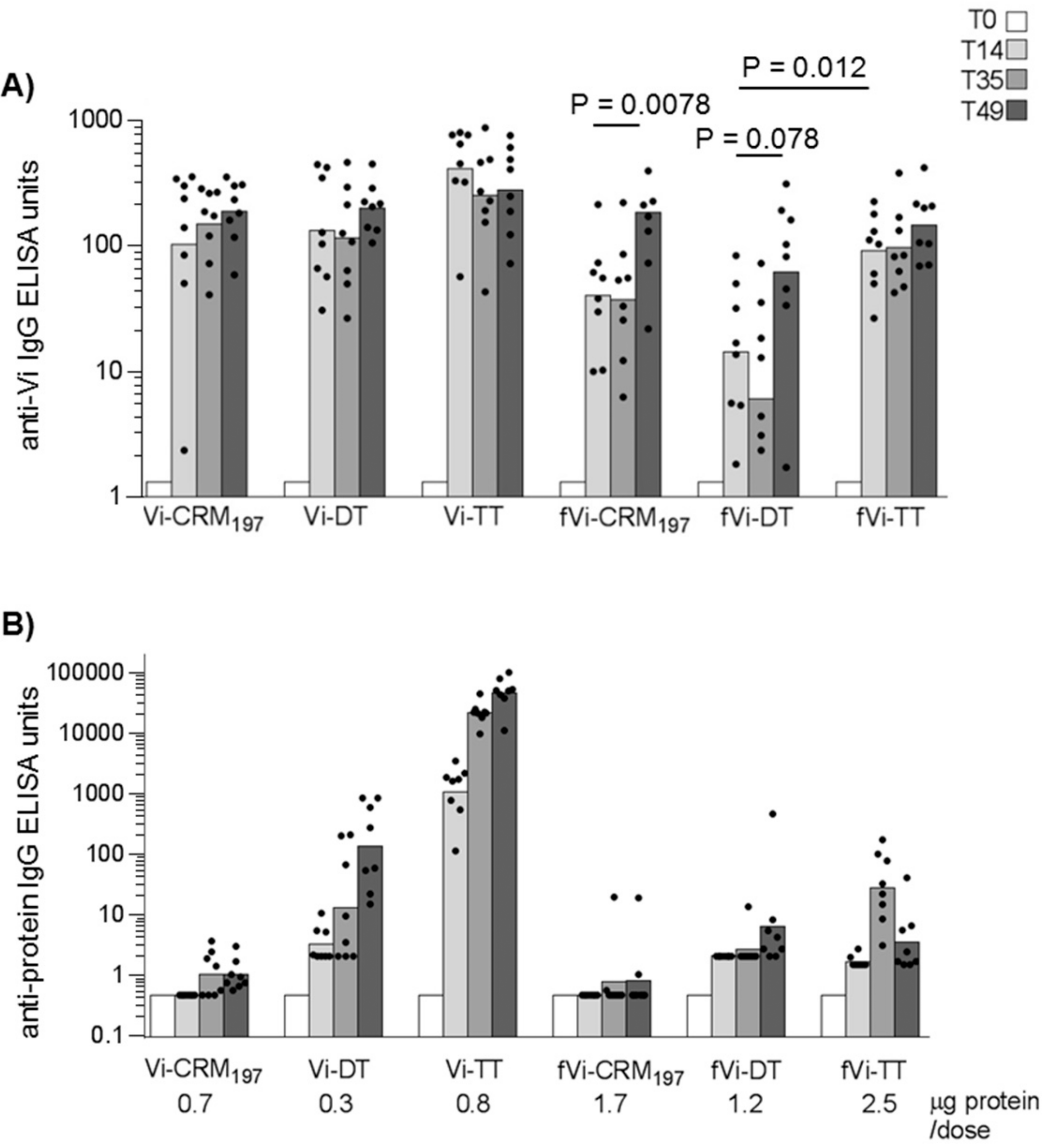
Influence of carrier protein on the immunogenicity of Vi conjugates in mice. Ten weeks old CD1 mice were immunized at days 0 and 35 with 1 μg Vi/dose. A) anti-Vi IgG ELISA titres and B) anti-carrier IgG ELISA titres. Bars represent the geometric mean ELISA units of the group in log scale, individual animals are represented by the scatter plots.

Mice were immunized to each receive 1 μg Vi/dose on days 0 and 35. All full-length Vi conjugates induced peak anti-Vi antibody responses 14 days after primary injection, with no significant augmentation of titres observed 14 days after secondary immunization (Fig 2A). Anti-Vi IgG responses were similar in mice receiving full-length Vi conjugates, independent of the carrier protein. The anti-Vi IgG titres induced by fVi-TT conjugate were significantly higher at day 14 after primary immunization than those observed after immunization with the fVi-DT conjugate (p = 0.012), but not with fVi-CRM_197_ (p = 0.41). Nevertheless, no significant differences in the anti-Vi IgG response were observed between fVi conjugates 14 days after secondary immunization (Fig 2A). Secondary immunization did not increase the titres of mice immunized with fVi-TT further, whereas the titres did increase after secondary immunization with fVi-DT (p = 0.078) and fVi-CRM_197_ (p = 0.0078) conjugates (Fig 2A). After two immunizations mice that had either the full-length or fVi conjugates all had similar anti-Vi IgG responses (Fig 2A).

All full-length conjugates induced an anti-carrier response higher than the corresponding fragmented Vi conjugates (Fig 2B), except for CRM_197_ for which the response was very low also with full-length Vi. This was the case despite mice immunized with fVi conjugates received more protein in the immunization (Fig 2B).

### Saccharide to protein ratio and conjugate cross-linking/size do not influence the anti-Vi IgG response

**Fig 3:**
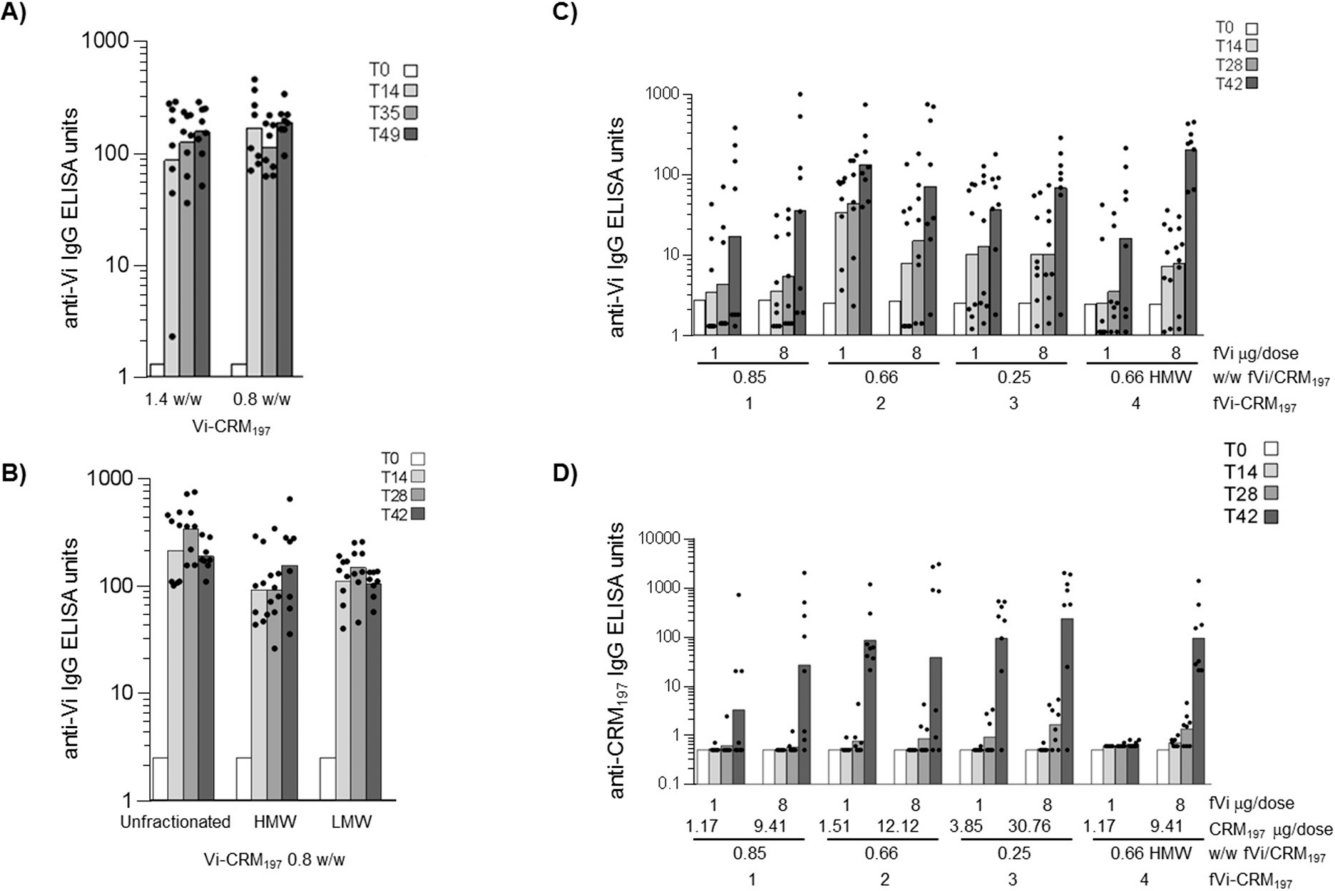
Influence of saccharide to protein ratio and conjugate size on: anti-Vi IgG response induced in mice by Vi-CRM_197_ (A and B respectively) and fVi-CRM_197_ conjugates (C); anti-CRM_197_ IgG response induced in mice by fVi-CRM_197_ conjugates (D). Ten weeks old CD1 mice were immunized at days 0 and 28 or 35 at 1 μg (A, B) and 1 or 8 μg (C) Vi/dose. Bars represent the geometric mean ELISA units of the group in log scale, individual animals are represented by the scatter plots. HMW and LMW indicate high molecular weight and low molecular weight conjugates.

The impact of saccharide to protein ratio and conjugate crosslinking/size on the immunogenicity of full-length (165 kDa) and fVi (avMW 43 kDa) conjugated to CRM_197_ was evaluated. The IgG response to full length Vi-CRM_197_ conjugates with a w/w Vi to protein ratio of 1.4 or 0.8 respectively (Table 2), but with a similar size by HPLC-SEC, was assessed by immunization in mice receiving 1 μg Vi/dose. These experiments showed that altering the ratio of Vi to protein did not influence the anti-Vi response (Fig 3A) nor the anti-protein IgG response, which were similar in both groups.

**Table 2:** Full-length and fragmented Vi-CRM_197_ conjugates differing for saccharide to CRM_197_ ratio or size.

Conjugate	PS concentration in conjugation mixture	PS to CRM_197_ ratio w/w in conjugation mixture	PS to CRM_197_ ratio w/w in purified conjugate
Vi-CRM_197_	2.5	1	1.37
	2.7	0.5	0.80
fVi-CRM_197_ 1	7.8	2	0.85
fVi-CRM_197_ 2	7.8	1	0.66
fVi-CRM_197_ 3	7.8	0.5	0.26
fVi-CRM_197_ 4	20	1	0.66

All purified conjugates showed less 20% free Vi and no detectable amounts of free protein.

Next we assessed whether separating out full-length Vi-CRM_197_ into higher molecular weight (HMW) and lower molecular weight (LMW) fractions altered the immune response. HMW and LMW fractions were collected after separation on a Sephacryl S1000 16 90 column in PBS. The HMW and LMW pools had the same Vi to CRM_197_ w/w ratio of 0.8 as the unfractionated conjugate, but with different sizes, likely related to differences in the level of crosslinking. HMW, LMW and unfractionated corresponding full-length Vi-CRM_197_ conjugate were characterized on a Superose 6 10/300 GL column (GE) eluting at 0.3mL/min with PBS, using DNA and NaN_3_ to calibrate the column. Kd values, defined as [(retention time of NaN_3_ –retention time of conjugate)/(retention time of NaN_3_ –retention time of DNA)], were calculated and determined to be 0.016 for the unfractionated conjugate and 0.021 for the LMW fraction. HMW conjugate eluted before DNA, used to define the void volume of the column.

Anti-Vi IgG titres induced, after both primary or secondary immunization, with either the HMW or LMW Vi-CRM_197_ fractions were comparable with those titres seen after immunization with the non-fractionated conjugate (Fig 3B).

In order to assess if immunization with fVi-CRM197 conjugates varying in the polysaccharide to protein ratio or conjugate size influences the anti-Vi IgG response (Table 2, Fig 3C), mice were immunized to receive 1 or 8 μg of fVi/dose. For all conjugates, we observed variability in anti-Vi antibody response among individual mice, with some non-responders. At both doses tested, there was no significant difference among the IgG response induced in groups immunized with the conjugates characterized by different saccharide to protein ratio and similar size (fVi-CRM_197_ conjugates 1, 2 and 3 in Table 2) (Fig 3C).

With the 8 μg fVi dose conjugates, the anti-CRM_197_ IgG response (Fig 3D), was minimal after one only injection, and then increases to a similar level for all the constructs post second dose, independent of the dose of carrier injected. With the 1 μg fVi dose conjugates, the anti-CRM_197_ IgG response induced by the conjugate with 0.85 w/w Vi to protein ratio was significantly lower (p = 0.047) than the response induced by the conjugate with 0.26 w/w ratio. Otherwise responses were similar.

**Fig 4:**
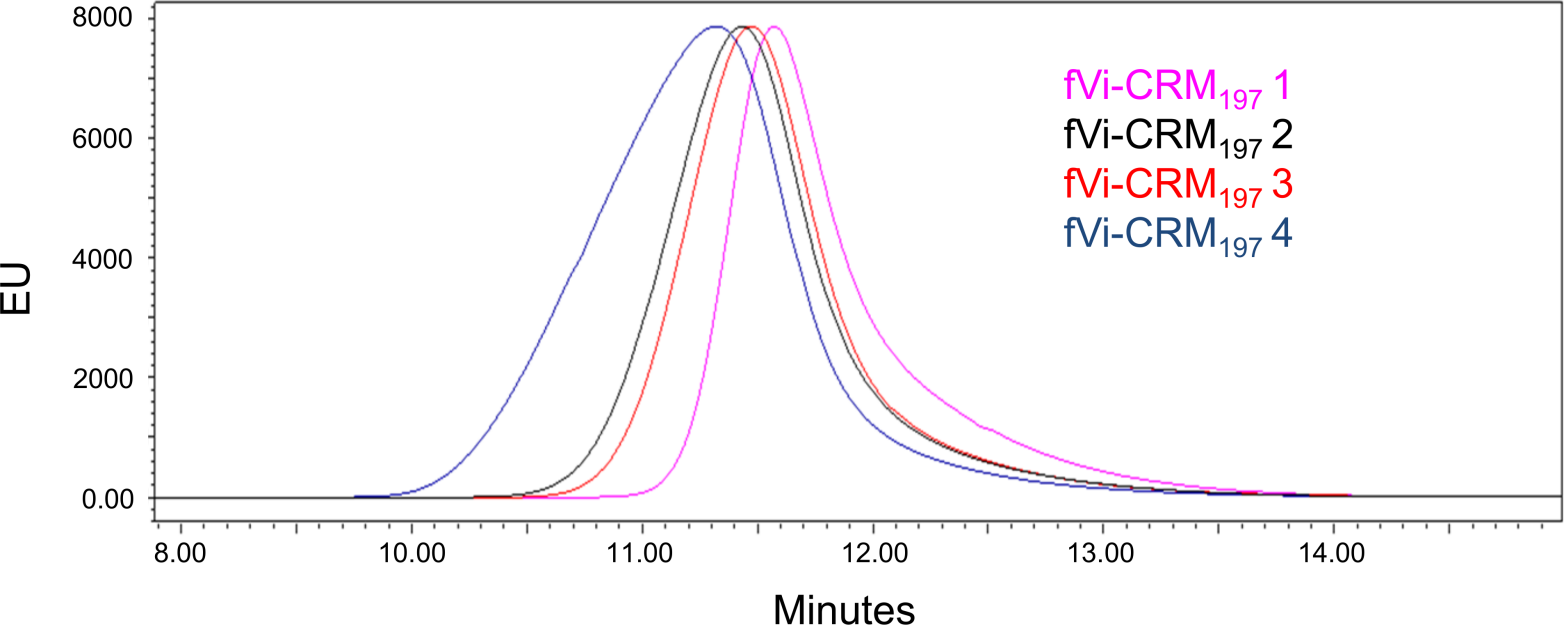
HPLC-SEC profiles (fluorescence emission detection) on TSK gel 3000 PWxl column (NaH_2_PO_4_ 100 mM, NaCl 100 mM, 5% CH_3_CN pH 7.2; 0.5mL/min) of fVi-CRM_197_ conjugates 1 (pink), 2 (black), 3 (red) and 4 (blue) (Table 2).

fVi-CRM_197_ conjugates that have the same fVi to protein ratio (0.66 w/w) but different size (fVi-CRM_197_ conjugates 2, having retention time of 11.3 min, and 4, with retention time of 11.45 min in Table 2 (Fig 4) induced similar anti-Vi IgG responses (Fig 3C). Also here, differences in geometric means among the groups are reflection of non/low-responders mice. At 1 μg Vi dose, the larger conjugate did not induce an anti-protein IgG response (Fig 3D). There was no statistically significant difference in the anti-CRM_197_ IgG response when the conjugates were tested at 8 μg Vi dose.

### The method of conjugation chemistry used does not influence the immunogenicity of the vaccine

**Fig 5:**
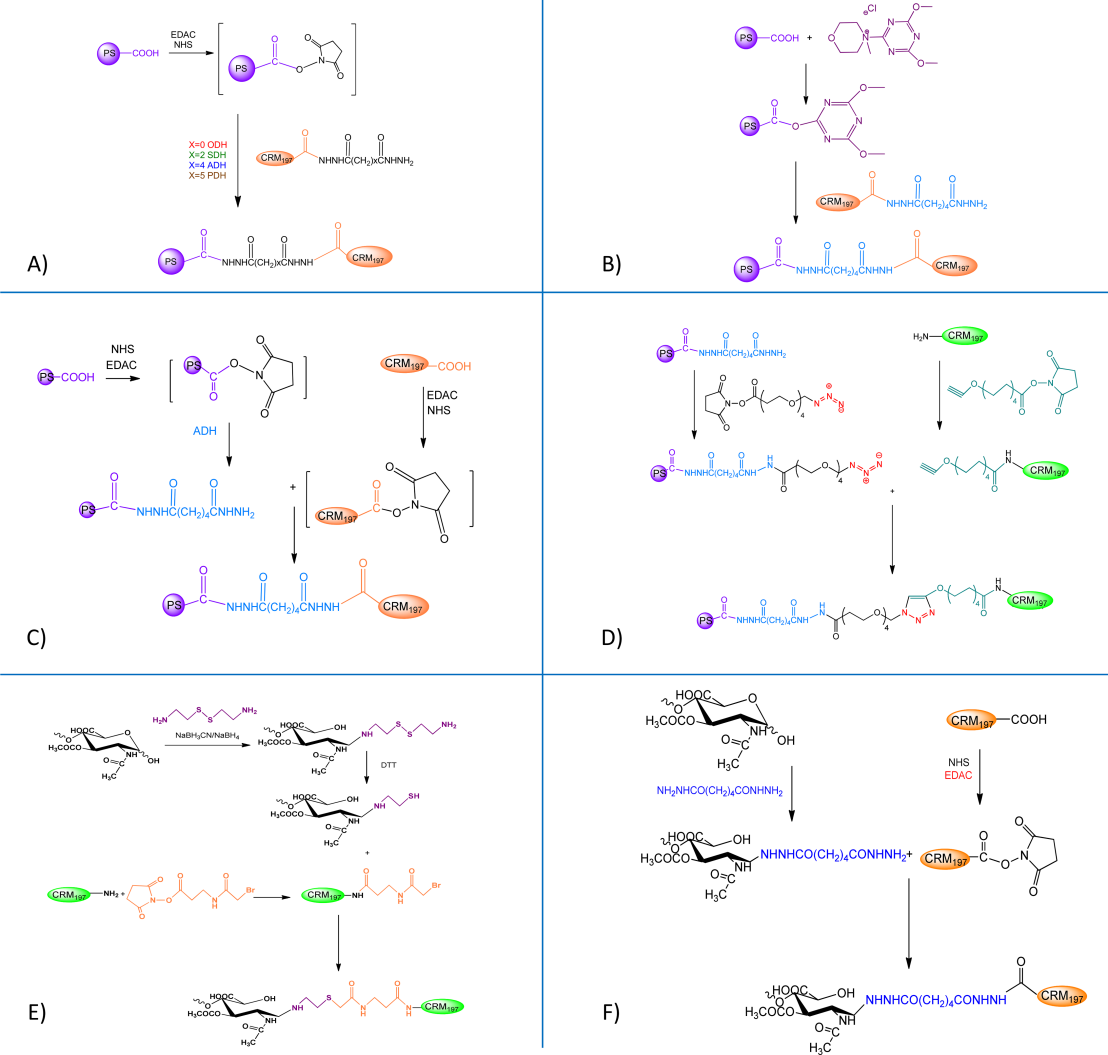
Conjugation scheme for the synthesis of A) fVi-CRM_197_ conjugates differing for the length of the spacer molecule introduced on CRM_197_ before conjugation to fVi; B) fVi(DMT-MM)CRMADH; C) fVi(ADH)-CRM_197_; D) fViADHN_3_-CRMalkyne; E) fVis(ADH)-CRM_197_; F) fVisSH-CRMSBAP (Table 3).

Different conjugation chemistries were tested to link fVi avMW 43 kDa to CRM_197_ as carrier protein. Linkers of differing length were introduced onto CRM_197_ (NH_2_NHCO(CH_2_)_x_CONHNH_2_, x = 0, 2, 4, 5). Then fVi was conjugated to these carrier proteins using the scheme shown in Fig 5A. By using same reaction conditions for CRM_197_ derivatization with ADH [10], an average of 9 linkers were introduced per molecule of protein with PDH (x = 5) and an average of 6–7 linkers with all the other linkers (Table 3). There was no correlation between the number of linkers per CRM_197_ molecule and linker length, or the saccharide to protein ratio of the corresponding conjugates (Table 3).

**Table 3:** Characteristics of the conjugates obtained using different conjugation strategies.

Conjugation scheme		Conjugate (n° CH_2_ in the linker)	fVi avMW	Component derivatised (activation degree)	Aminoacids activated on CRM _197_	fVi to CRM_197_ w/w ratio	fVi to CRM_197_ molar ratio	% free fVi^*^
**Random**	1A	fVi-CRMODH(x = 0)	43	CRM_197_(7 linkers)	Asp/Glu	0.61	na	5.67
		fVi-CRMSDH(x = 2)		CRM_197_(6 linkers)	Asp/Glu	0.78	na	25.2
		fVi-CRMADH(x = 4)		CRM_197_(6 linkers)	Asp/Glu	0.59	na	<20
		fVi-CRMPDH(x = 5)		CRM_197_(9 linkers)	Asp/Glu	0.46	na	0.3
	1B	fVi(DMT-MM)CRMADH		CRM_197_(6 linkers)	Asp/Glu	0.51	na	13.5
	1C	fVi(ADH)-CRM_197_		fVi(22% RU)	Asp/Glu	1.63	na	<20
	1D	fViADHN_3_-CRMalkyne		CRM_197_(12 linkers) fVi(22.5% RU)	Lys	0.34	na	<20
**Selective terminal**	1F	fVi_s_(ADH)-CRM_197_	8.6	fVi(1linker/chain)	Asp/Glu	0.23	1.53	<20
	1E	fVi_s_SH-CRMSBAP		CRM_197_(9 linkers) fVi(1 linker/chain)	Lys	0.28	1.91	<20

RU = repeating units, na = not applicable, *no free CRM_197_ detected in all the conjugates.

An additional conjugate was synthesized using DMT-MM to activate COOH groups on fVi, instead of EDAC/NHS, before linkage to CRMADH (Fig 5B). This reagent has been tested as an alternative coupling reagent to carbodiimide, and has a shorter activation step than using EDAC/NHS [23, 24]. The resulting conjugate had same structure and similar Vi to protein ratio than fVi-CRMADH (Table 3).

Another conjugate was synthesized by derivatizing fVi with ADH instead of CRM_197_ before performing conjugation (Fig 5C). Derivatization of the PS is preferable to the derivatization of the protein, as it avoids performing multiple reaction steps on CRM_197_, an expensive component of the vaccine. The conjugate obtained (fVi(ADH)-CRM_197_) was characterized by a 3-fold higher Vi to protein ratio than fVi-CRMADH (Table 3).

**Fig 6:**
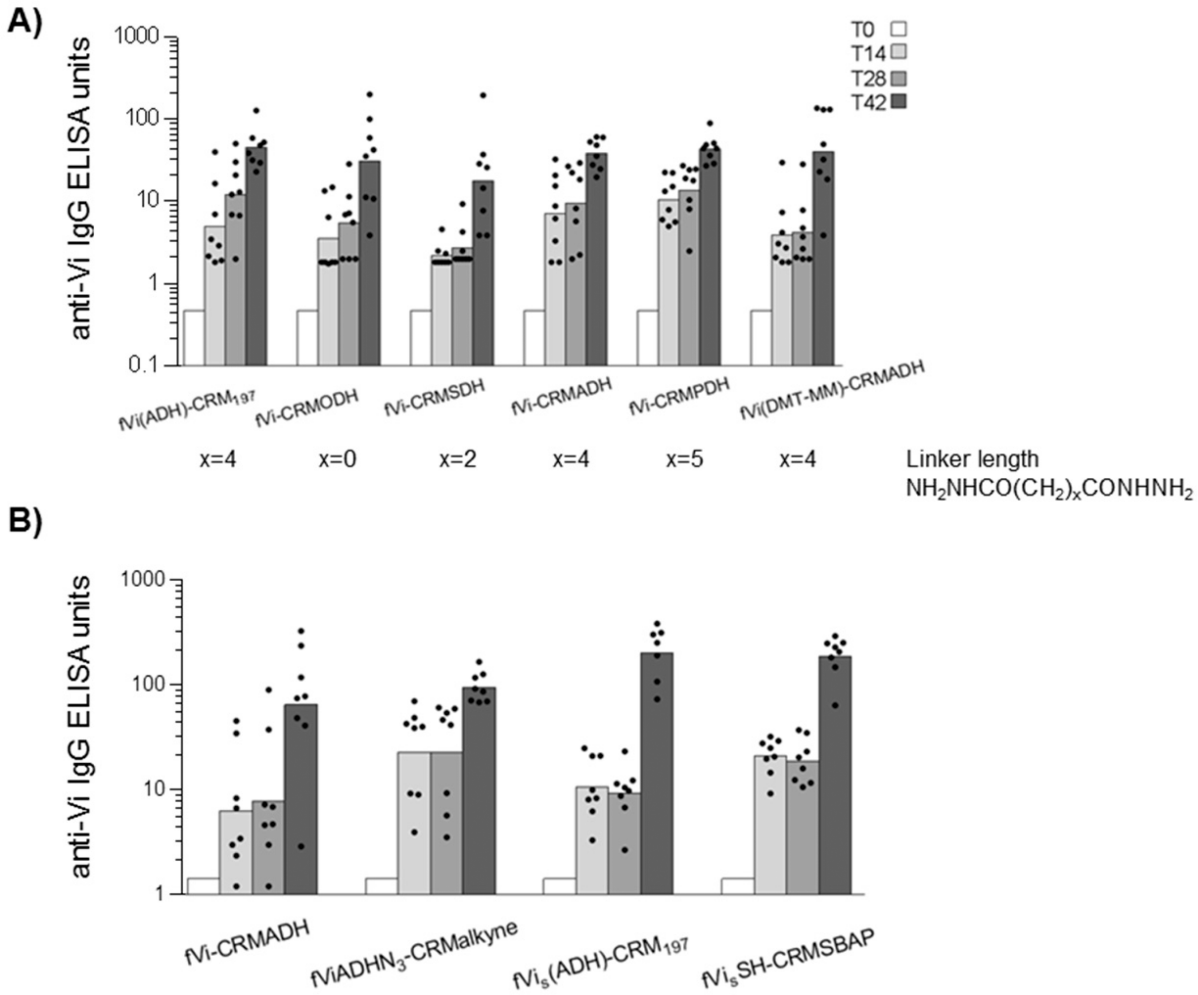
Influence of conjugation chemistry (Table 3) on the immunogenicity of fVi-CRM_197_ conjugates in mice: anti-Vi IgG ELISA titres. Five weeks old CD1 mice were immunized at days 0 and 28 at 1 μg Vi/dose in two separate studies (A and B). Bars represent the geometric mean ELISA units of the group in log scale, individual animals are represented by the scatter plots.

Although after immunization with these different conjugates there was no significant difference among the anti-Vi IgG responses induced, those obtained using the longer linker between the saccharide and protein moiety (ADH or PDH) gave a more consistent immune response (Fig 6A). fVi(ADH)-CRM_197_ conjugate was characterized by a higher Vi to protein ratio than all the other conjugates. The lack of difference in anti-Vi IgG antibody response induced compared to the other conjugates supports our finding that the saccharide Vi to protein ratio is not a critical parameter for the immunogenicity of fVi-CRM_197_ conjugate vaccine (Fig 3C). No difference among the groups was also found in terms of anti-CRM_197_ IgG response.

We then compared fVi-CRMADH with a conjugate obtained by random activation of fVi, but targeting lysine amino acids on CRM_197_ instead of carboxylic groups (fViADHN_3_-CRMalkyne, Fig 5D; Table 3). In this experiment we also included two further conjugates prepared by selective terminal linkage of fVi chains to CRM_197_, one targeting carboxylic groups (fVi_s_(ADH)-CRM_197_) and the other one lysine residues on CRM_197_ (fVi_s_SH-CRMSBAP) (Fig 5E and 5F; Table 3). The selective conjugates were prepared with fVi with a lower average MW of 8.6 kDa, as selective chemistries did not work with the longer Vi chains with an avMW 43 kDa. These conjugates were then tested in mice (Fig 6B), and the results show different conjugation chemistries had no impact on the anti-Vi IgG responses detected (Fig 6B). However, two weeks after the second injection, the anti-CRM_197_ response induced by fVi_s_(ADH)-CRM_197_ (mean value of 330.7) was significantly higher than the response induced by the random conjugates fVi-CRMADH (mean value of 20.7) and fViADHN_3_-CRMalkyne (mean value of 32.1), with p values of 0.0002 and 0.01 respectively.

## Discussion

It is well known that several parameters can affect the immunogenicity of glycoconjugate vaccines [8]. Here we used a systematic approach to look at the impact of multiple variables on the immunogenicity of a glycoconjugate vaccine against *S*. Typhi.

The length of the Vi chain linked to the carrier influenced the kinetics of antibody induction to the PS. Reflecting what is observed in humans [12, 13], full-length Vi-CRM_197_ induced high anti-Vi IgG responses in mice after one immunization, with no anamnestic response following a second immunization. In contrast to the full-length Vi conjugate vaccine, a fVi-CRM_197_ conjugate could boost specific anti-Vi IgG antibody levels following a second immunization, although the final magnitude of the response was not greater than that observed with the full-length Vi conjugate. This could be related to the ability of full-length Vi to act in a T-independent manner, which may not be completely lost after conjugation to the carrier protein. Also, high titres of circulating anti-Vi antibodies could inhibit the ability of the conjugate to boost the response.

Conjugate size did not impact the anti-PS specific IgG response induced in mice. In contrast, An et al. found that, for Vi-DT conjugates, the larger and more cross-linked the conjugate, the higher the anti-Vi response induced after one injection [15]. Wessels et al. also showed that larger and more cross-linked GBS type III-TT conjugates induce higher IgG anti-saccharide responses [25]. Findings are difficult to generalize for all glycoconjugate vaccines. Different antigens and differences in other conjugation variables, such as carrier protein, can give different results.

Theoretically, any protein containing peptides that can be presented through MHCII and recognized by CD4+ T cells can be used as a carrier protein. Nevertheless, few proteins have been used as carriers in licensed glycoconjugate vaccines to date, with DT, TT and CRM_197_ used for Hib, pneumococcal and meningococcal conjugate vaccines [8, 26]. TT and DT have traditionally been used because of safety data collected with tetanus and diphtheria vaccination. CRM_197_ does not require chemical detoxification, facilitating its production and resulting in more standardized preparations [27]. There has been particular interest in the influence of the carrier protein on the immunogenicity of conjugate vaccines, often to the exclusion of other parameters. In the context of Vi conjugate vaccines, several proteins, including *r*EPA, DT, TT, CT, the B subunit of the heat-labile toxin of *E*. *coli*, recombinant OMP of *Klebsiella pneumoniae* (*r*P40) and iron-regulated OMP of *S*. Typhi have been tested as carriers [6, 10, 16, 18, 20, 28–31]. Two studies have investigated the impact of carrier protein on the immunogenicity of Vi conjugates in mice. Both studies found no effect of the carrier protein (CRM_197_, TT, DT or *r*EPA) on the immunogenicity of full-length Vi conjugate vaccines obtained by EDAC random chemistry with ADH linker [10, 16].

In our study, we have confirmed that full-length Vi conjugates induce similar anti-Vi IgG responses independent of the carrier protein used in the conjugation. The same was observed for fragmented Vi conjugates when administered in two doses 28 days apart. fVi-CRM_197_ and fVi-DT gave a peak response after one immunization, similar to the response seen for full-length Vi conjugated to TT, but did not give a booster response after the second injection. This differs from the response seen after immunization with fVi-TT. The different behavior of TT compared to CRM_197_ and DT could be related to the larger size of TT. Higher primary PS-specific antibody responses elicited by TT conjugates, compared to DT or CRM_197_ as carrier proteins, has been shown also in other studies [32, 33].

The full-length conjugates induced anti-carrier responses higher than the corresponding fragmented conjugates, except for CRM_197_ conjugates for which the response was low with both full-length and fragmented Vi. This was unexpected because of the higher amount of protein administered with fragmented conjugates and because longer Vi chains might be expected to potentially mask more of the protein moiety [18]. However, in a study comparing pneumococcal polysaccharide and oligosaccharide TT conjugates, an inverse correlation was found between protein-specific IgG responses and the protein dosage administered for polysaccharides but not for oligosaccharides [34]. Furthermore, in a study comparing long and short polyribosylribitol phosphate polysaccharide conjugated to TT carrier protein, the anti-TT antibody response was higher for the longer PS conjugate, consistent with our results. It has been suggested that during conjugation smaller saccharides could have a major impact on protein epitopes compared to longer polysaccharides [35]. In a recent analysis of structure-antibody recognition relationships in nine licensed polysaccharide-TT conjugate vaccines, it was found that recognition of the carrier epitopes was not necessarily hampered by the size of the conjugate or the saccharide loading [36]. An alternative possible explanation is that longer Vi chains may better stabilize the protein resulting in the higher anti-carrier antibody levels seen.

The use of shorter Vi chains can offer advantages for vaccine manufacture: the conjugation reaction can be performed with a higher degree of control and better consistency due to the higher solubility of shorter PS. Yields of conjugate (expressed in terms of the Vi in the conjugation mixture) are higher and the product is easier to sterilize by filtration. It is also easier to purify (particularly from unreacted PS) and to characterize. With this in mind, we investigated the impact of conjugation chemistry on shorter Vi chains and with CRM_197_ carrier protein. We tested linking Vi by random and selective conjugation chemistries, targeting different amino acids on CRM_197_ [37] and using linkers of different length, and found there was no major impact on the anti-Vi IgG response in mice after immunization. In contrast to our findings, the only other study investigating the impact of conjugation chemistry on the immunogenicity of full-length Vi conjugate vaccines found that Vi-*r*EPA conjugates induce higher anti-Vi antibody levels, both in animals and in humans, when EDAC/ADH chemistry was used instead of cystamine/SPDP [17].

Selective chemistries produce better-defined structures, avoiding chemical modification of the saccharide chain, compared to random approaches, which result instead in high MW, cross-linked and rather undefined and heterogeneous structures. However, we found difficulty applying terminal selective chemistries to Vi chains.

During our study we tried to investigate single parameters in parallel, by introducing only one change per conjugate. However, the saccharide to protein ratio in the resulting conjugates was influenced by the saccharide chain length, protein and conjugation chemistry used and so was difficult to control. Nevertheless, both for full length and shorter Vi chains of 43 kDa we showed there was no major impact of the saccharide to protein ratio on the immunogenicity of the conjugate in mice.

Few studies have looked at the impact of Vi to protein ratio on the immunogenicity of Vi conjugate vaccines [11, 15]. Rondini et al. showed that for Vi-CRM_197_, a different Vi to protein ratio did not impact greatly on the immune response [11]. In fact, a Vi to CRM_197_ ratio of 10.1 was suboptimal compared to conjugates with a Vi to CRM_197_ ratio of 0.9 and 2.1, only at the lower dose of 0.125 μg Vi in the range 0.125–8 μg tested, while no differences were observed at the higher doses tested. An et al. [15] showed instead that the amount of DT conjugated to Vi influences the magnitude of the response to the PS. The more DT bound (range of Vi/DT w/w tested of 0.7–7.1), the greater the anti-Vi IgG response following two injections. In their study, the conjugates tested had differing Vi to protein ratios and levels of cross-linking. Thus it is difficult to identify which parameter in their study was impacting most on the immune response.

In conclusion, from this study, the saccharide chain length and the carrier protein can modulate the immunogenicity of Vi conjugate vaccines more than the other parameters assessed. The influence of these two variables on the immunogenicity is interconnected, as shown by the different behavior of fragmented Vi when linked to CRM_197_ or TT. Therefore, when generating novel Vi glycoconjugates it may be optimal to focus on these two elements rather than the chemistries used to link the molecules together. It is interesting to note that Vi-CRM_197_ and Vi-*r*EPA behaved differently when tested in infants [13, 38]. The two conjugates not only differed in the carrier used, but also in the source and size of Vi polysaccharide component and the combination of both these factors could have contributed to the findings.

Although conjugate vaccine design is clearly an important factor in promoting efficient immunity, other factors will contribute to the overall success of vaccination. Included in these are the intrinsic capacity of a population to respond optimally to a vaccine. For instance, in regions that have a high incidence of infections that are endemic, multiple reports have indicated that they induce lower responses to vaccination than populations in developed countries [13].

Our conclusions are based on results in adult mice and it would be important to assess how these vaccines would work in younger mice and how this may translate into humans. Also non-conjugated Vi induces IgG in adult mice after immunization. Nevertheless, the magnitude of this IgG response tends to be lower than the response induced by the conjugates and the IgG isotype induced in the response differs, with IgG3 being the major IgG isotype observed[39].

The approach used here can be extended to other conjugate vaccines in order to identify critical conjugation parameters and select their optimal combination to result in improved glycoconjugate vaccines in terms of production and potential efficacy.

## Supporting information

S1 File (PDF)
